# Surgical treatment of popliteomeniscal fascicles tears is associated with better patient-reported outcome measures. A systematic review and meta-analysis

**DOI:** 10.1007/s00590-023-03645-4

**Published:** 2023-07-23

**Authors:** Virginia Masoni, Fortunato Giustra, Francesco Bosco, Lawrence Camarda, Giuseppe Rovere, Veronica Sciannameo, Paola Berchialla, Alessandro Massè

**Affiliations:** 1 Department of Orthopaedics and Traumatology, University of Turin, CTO, Via Zuretti 29, 10126 Turin, Italy; 2 Department of Orthopaedics and Traumatology, Ospedale San Giovanni Bosco di Torino - ASL Città di Torino, Turin, Italy; 3 Department of Orthopaedics and Traumatology (DiChirOnS), University of Palermo, Palermo, Italy; 4 Department of Orthopaedics and Traumatology, Fondazione Policlinico Universitario A. Gemelli IRCCS-Università Cattolica del Sacro Cuore, Rome, Italy; 5 Department of Clinical and Biological Sciences, University of Turin, Turin, Italy

**Keywords:** Popliteomeniscal fascicles, Locking, Figure-4, Surgery, PROMs

## Abstract

**Purpose:**

Popliteomeniscal fascicles (PMFs) are a component of the popliteal hiatus complex in the knee, and their injury primarily affects young athletes participating in sports activities involving twisting movements. The identification of PMFs tears presents a challenge, often accompanied by lateral pain and a locking sensation. The objective of this systematic review (SR) and meta-analysis is to enhance the suspicion and recognition of PMFs tears, aiming to facilitate the treatment of this condition, particularly in symptomatic young patients.

**Methods:**

A comprehensive search, focused on studies examining PMFs injuries and their treatment, was conducted in four databases, PubMed, Scopus, Embase, and Web of Science. The ROBINS-I tool was used to evaluate the risks of bias. The PRISMA flow diagram was used to conduct the research and select the included studies. A meta-analysis was conducted for the Lysholm score, the Tegner Activity Scale, and the subjective IKDC score. The present SR and meta-analysis was registered on PROSPERO.

**Results:**

Five clinical studies were included in the final analysis, comprising 96 patients. All the patients underwent a preoperative MRI assessment and a diagnostic arthroscopy to detect the PMFs tears, with a subsequent surgical procedure either open or arthroscopically performed. Surgery was associated with the resolution of symptoms. A statistically significant improvement in the Lysholm score (*p*: 0.0005) and the subjective IKDC score (*p*: 0.003) after the surgical procedure with respect to the preoperative evaluation was found.

**Conclusion:**

This SR and meta-analysis showed a significant improvement in the Lysholm score and subjective IKDC score following surgery for PMFs tears. However, controversy persists regarding the optimal surgical approach, with current literature favoring arthroscopic procedures.

**Level of evidence:**

IV.

## Introduction

Popliteomeniscal fascicles (PMFs) are part of the posterolateral complex (PLC) of the knee, and specifically, they have been associated with the popliteal hiatus complex [1–4]. There are three PMFs mentioned in the literature, the anteroinferior (aiPMF), the posterosuperior (psPMF), and the posteroinferior (piPMF). As grossly defined, they connect the lateral meniscus to the popliteal hiatus [3–5]. Concerning a more detailed description, the aiPMF creates the floor while the psPMF is the roof of the popliteal hiatus, respectively [1–5]. In literature, there has been some debate regarding the number of the PMFs, since the piPMF, described by Terry and LaPrade as formed from the aponeurosis of the popliteus muscle [3], is inconstant between individuals [1–5]. Peduto et al. identified it through MRI arthrography only in 40% of specimens [6]. The PMFs are important stabilizers of the lateral meniscus, and their damage is one of the causes of a hypermobile lateral meniscus (HLM) [1, 2, 4, 7, 8]. PMFs injuries occur mainly in athletes performing sports with sudden changes of direction, such as dancers, football players, and wrestlers [9]. Identifying PMFs tears is challenging both from a clinical point of view and magnetic resonance imaging (MRI) assessment [4, 5, 9–11]. This is because they rarely occur as isolated lesions but are often associated with anterior cruciate ligament (ACL) or PLC knee injuries [1, 9, 11–13]. As mentioned by Temponi et al., nearly 20% of patients with an ACL lesion have some injury to the PLC when evaluated by MRI, including PMFs tears [13]. From a clinical point of view, lateral pain and a sensation of locking or snapping are the main symptoms [4, 9, 11]. LaPrade et al. introduced a clinical test, the figure-4 position, reproducing pain on the lateral knee due to entrapment of the lateral meniscus into the joint in case of damage to the PMFs [5]. Concerning imaging, MRI is widely used, but some authors [2, 5] reported an initially apparently normal preoperative MRI. For this reason, the gold standard for the diagnosis is an arthroscopy with direct visualization of PMFs tears and probing of the lateral meniscus mobility [5, 9, 14]. Subsequently, when the diagnosis of PMFs tears is accomplished in symptomatic patients, evidence suggests surgically managing it [4, 5, 9].

Numerous surgical techniques have been proposed, from the open repair by LaPrade and Konowalchuk [5] to the all-inside arthroscopy suture [8, 11, 15]. The superiority of one technique over the others is under debate in the literature, with the resolution of the symptoms in all the cases. [5, 7–9, 15]. Indeed, due to the scarcity of studies on this topic, only a few authors have evaluated the outcomes with knee functional scores [7, 8, 11, 15].

This systematic review (SR) and meta-analysis helps to achieve a correct diagnosis and analyzes the postoperative outcomes through patient-reported outcome measures (PROMs) as the Lysholm score, the Tegner Activity Scale, and the subjective International Knee Documentation Committee (IKDC) score. The purpose of this study was to underline the importance of recognizing PMFs tears to treat this condition, especially in symptomatic young patients, since surgery appears resolutive for the symptoms.

## Material and methods

### Research question

The current literature regarding PMFs was explored by two authors (VM and FB) who performed the research independently and reviewed the articles autonomously to avoid possible bias. In cases of disagreement, a third author (FG) was consulted. The Preferred Reporting Items for Systematic Reviews and Meta-Analyses (PRISMA) flow diagram was used to conduct the research and to select the final studies included in the present SR and meta-analysis [16]. The Patient, Intervention, Comparison, Outcomes, and Study (PICOS) design was used to answer clinical questions; (P) patients with diagnosis of PMFs tears; (I) PMFs tears undergoing surgical procedure; (C) comparison between pre- and postoperative PROMs and symptoms; (O), postoperative clinical, functional (PROMs), and radiographic outcomes after surgical procedure when available compared with pre-surgical setting; (S) study design, retrospective studies.

### Search strategy and study selection

**Fig. 1:**
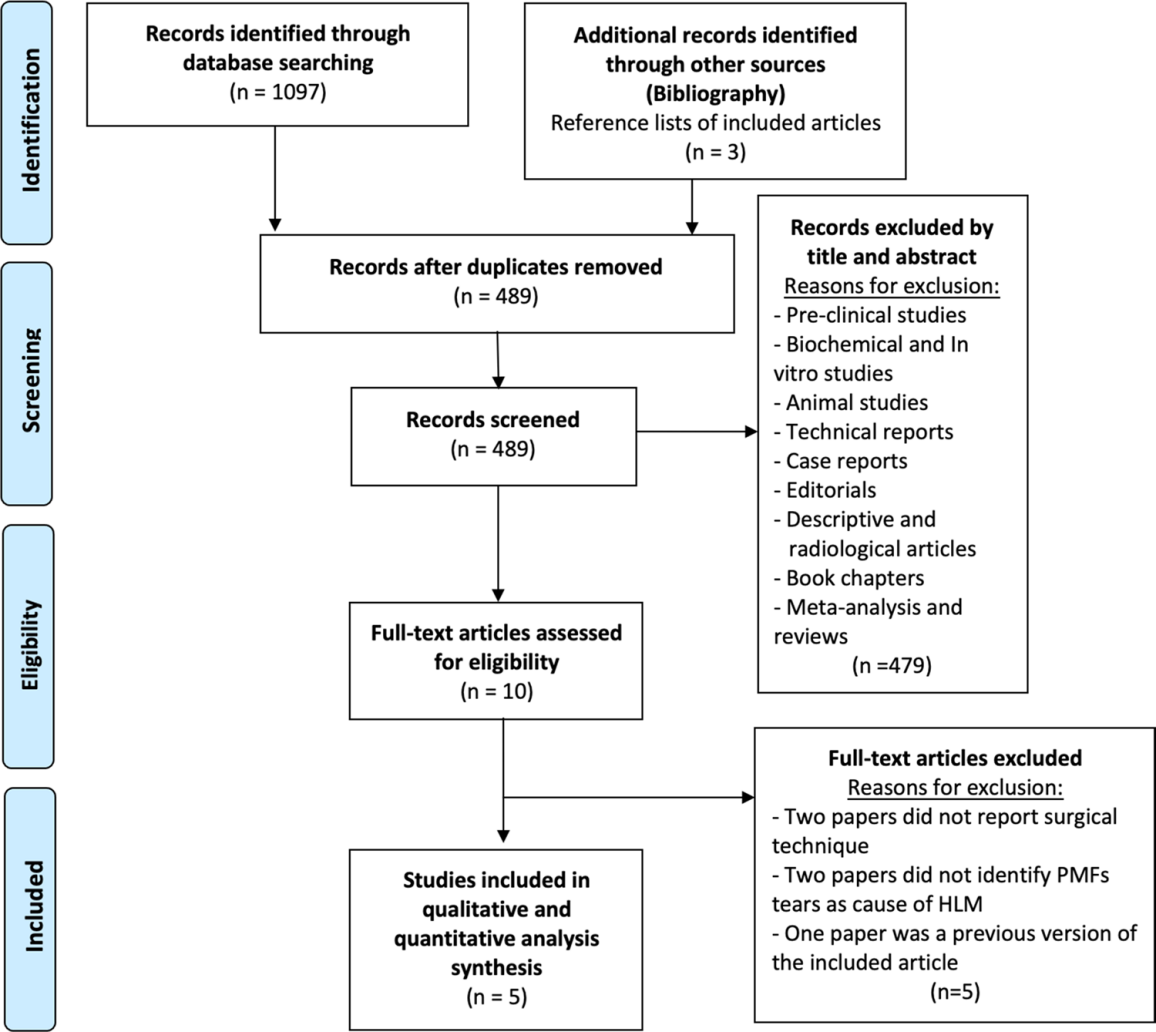
Preferred reporting items for systematic reviews and meta-analyses (PRISMA) flow diagram of the studies included in the analysis. *PMFs* popliteomeniscal fascicles; *HLM* hypermobile lateral meniscus

A comprehensive search in four databases, PubMed, Scopus, Embase, and Web of Science, was performed with the following MeSH terms: [((popliteomeniscal fascicles) OR (lateral meniscus) OR (posterolateral corner) OR (popliteal hiatus)) AND (tears) AND (surg*)]. The search ended on the 1st of April 2023, with the most recent SR regarding this topic dating to 2021 [9]. A total of 1097 studies were identified through the comprehensive search. After eliminating the duplicates, 489 were considered. Of these, 479 were excluded after examining the title and the abstract. Further, three studies were added from the references of the included studies. By evaluating the full text, five studies were included for the final qualitative and quantitative analysis [5, 7, 8, 11, 15]. Two of them were included in the previous SR [5, 7], while two articles were de novo included since they were both published outside the temporal window of the previous SR [8, 15]. One of the articles included was divided into two sections: a cadaveric and a clinical human part. Thus, only the human investigation was considered in the analysis [15]. One of the articles included an updated version of a study in the previous SR [11]. The PRISMA flow diagram is shown in Fig. 1.

### Inclusion and exclusion criteria

The articles included were written in English and published between January 2000 and April 2023, concerning the human subjects only presenting with a tear of PMFs undergoing a surgical procedure. Biochemical studies, in vitro studies, animal studies, case reports, editorials, technical reports, descriptive and radiological articles, book chapters, pre-clinical studies, meta-analyses, and review articles were excluded from the analysis. Only studies reporting a preoperative imaging assessment through MRI and a diagnostic arthroscopy were included. Papers not reporting the surgical procedure adopted and the outcomes through symptoms, clinical examination, or PROMs were excluded. The associated lesions reported were not considered as either inclusion or exclusion criteria. After a meticulous investigation, data from the selected studies were inserted in Excel spreadsheets by two authors independently (VM and FB).

### Data extraction

Data extracted from the five studies included in the analysis were authors and publication year, the Levels of Evidence (LoE) of the study, the number of patients, the age, the sex, the follow-up time, the duration of symptoms before surgery, the mechanism of injury, the level of activity pre- and post-injury. Pre and postoperatively were analyzed: the symptoms reported by the patients, the clinical evaluation with physical examination, the MRI assessment, and the PROMs as the Lysholm score, the Tegner Activity Scale and the subjective IKDC score. The surgical description, including the diagnostic arthroscopy, the surgical procedure adopted, the suture type, the associated lesions, and the intra- and postoperative complications, were examined. In a separate spreadsheet, the rehabilitation protocol was summarized. Two professional statisticians (VS and PB) performed the data analysis.

### Quality evaluation

**Fig. 2:**
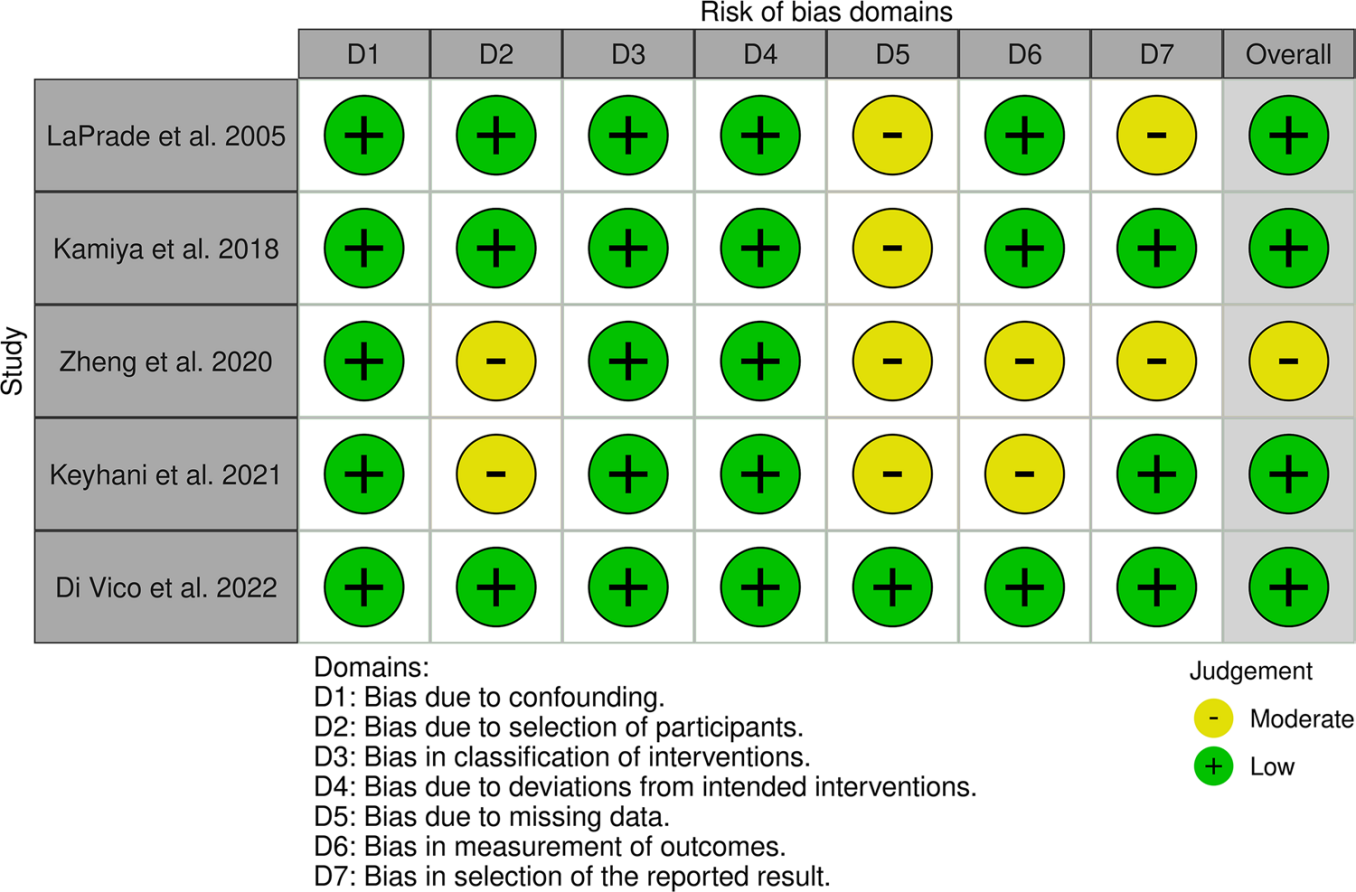
Assessment of the risk of bias of the individual studies included in the analysis according to the ROBINS-I tool (Risk of Bias In Non-randomized Studies of Interventions)

Eligible articles were assessed according to the Levels of Evidence (LoE) of the Oxford Centre for Evidence-Based Medicine 2011 [17]. All the articles included in the final analysis were LoE IV. The quality of the studies was evaluated using the Risk of Bias In Non-randomized Studies of Interventions (ROBINS-I) tool [18, 19] (Fig. 2). Two authors (VM and FB) utilized this tool, and a third author (FG) was consulted in cases of uncertainty or disagreement. Any potential controversies were resolved through discussion. All the authors have made a substantial contribution to the conception and design of the study, the acquisition of the data, the drafting of the article, and the final editing. All the authors approved the final version of the article. This SR was registered on the International Prospective Register of Systematic Reviews (PROSPERO) [20].

### Functional outcome scores evaluation—PROMs

Three PROMs were used to assess the functional outcomes. The Lysholm score is 100 points-scoring, with higher scores representing a better functional status and comprising eight items: need of support when walking, limp, locking, pain, instability, swelling, stair-climbing capacity, and squatting [21]. The Tegner Activity Scale evaluates the capability to practice sports and work. It consists of a one-item score ranging from 0 to 10, where 10 stands for the ability to perform competitive sports [22]. The subjective IKDC score evaluates three main aspects: sports activities, symptoms and knee function and ranges from 0 to 100, with higher scores representing better functional outcomes [23].

### Data analysis

The Lysholm score, the Tegner Activity Scale and the subjective IKDC score were considered for the meta-analysis.

Random effect estimates are computed to generate summary measures for continuous outcomes, and inverse variance weighting is employed for pooling. Mean change from pre- to postoperative scores serves as the summary statistics while the 95% Confidence Interval (95% CI) for individual studies is established according to the standard normal distribution. Meanwhile, the 95% CI of the random effect estimate is based on the standard normal quantile rule [24]. In the computation of the standard deviation of the pre-post change in each study, a correlation coefficient between pre-post values of 0.7 is assumed. When only min and max values were available, the standard deviation (SD) was computed as (max–min)/4, assuming a normal distribution. The between-study variance τ2 was calculated via the Restricted maximum-likelihood estimator [25], and the 95% CI was calculated via the Q-profile method [26]. The Cochran's Q test and the Higgins I2 statistics are utilized to estimate the heterogeneity between studies. Values of I2 ranging from 0 to 24.9%, 25–49.9%, 50–74.9%, and > 75% indicate no, low, moderate or high heterogeneity, respectively. Statistical analyses were performed using the R software, version 4.2.1 [27] and the meta-R package [28].

## Results

A total of 96 patients (62 males and 34 females) were included in the SR and meta-analysis. All the patients underwent a preoperative MRI assessment and a diagnostic arthroscopy to detect the PMFs tears, with a subsequent surgical procedure either open or arthroscopically performed. Patient demographics, preoperative evaluations as the clinical examination, the symptoms, the mechanism of injury, the level of activity and MRI assessment, as well as the follow-up time and the duration of symptoms before surgery, are reported in Table 1. The surgical technique adopted, the type of suture and the associated lesions are reported in Table 2. The postoperative assessment, including the physical examinations, the symptoms, the PROMs, the complications, the MRI assessment, and the return to the previous activity, are listed in Table 3. Postoperative protocols are resumed in Table 4.

**Table 1:** Patient demographics and preoperative characteristics with associated study details

Authors and Publication year	LoE	Study type	Patients sample size, N	Age (years old)	Sex, N	Follow-up time**	Time from injury to surgery (months)		Mechanism of injury, (N)	Level activity pre-injury, (N)	Clinical symptoms, (N)	Clinical evaluation and physical examination, (N)	Imaging evaluation preoperatively
				Mean ± SD (range)	M	F	Mean ± SD (range)	Mean ± SD (range)					
LaPrade et al. 2005 [5]	IV	RS	6	26.7 ± 4.6 (19–33)	4	2	≃ 3 years (2.5–5 years)	5.7 ± 2.4 (2–9)	Takedown– Twisting in wrestling (2); Twisting (3); Twisting in football (1)	Professional wrestler (2); Football player-not specified the level (1)	Pain	Figure-4 test positive in all the patients	MRI
Kamiya et al. 2018 [7]	IV	RS	20	37.7 (19–63)	9	11	37.0 (24–68)	25.3 (0.3–60)	–	–	Locking	Apprehension in performing valgus and deep flexion of the knee + external rotation of the tibia. *	Virtual load 3D-MRI
Zheng et al. 2021 [15]	IV	RS	36	27.8 (19–42)	25	11	21.1 (15–24)	–	Trauma not specified	–	–	*	MRI
Keyhani et al. 2021 [8]	IV	RS	17	34.0 ± 6.0 (18–42)	10	7	≃ 3.5 years (3–5 years)	At least 6 months of conservative treatment	–	–	Pain, locking or snapping	–	MRI
Di Vico et al. 2022 [11]	IV	RS	17	22.0 ± 3.6 (14–35)	14	3	68.0 ± 24.0 (49–84)	16.0 ± 13.6 (2–28)	–	Semi-professional sport activity (7); Recreational sport activity (10)	Locking and popping (17), limping (9), tenderness to palpation of lateral compartment (17)	Figure-4 test positive in all the patients	MRI

*LoE* level of evidence; *RS* retrospective studies; *N* number; *SD* standard deviation; *M* male; *F* female

–: Not reported/not mentioned in the paper; *MRI* magnetic resonance imaging

*Described as recorded by the authors, but no data reported in the paper

**Reported in months, otherwise if reported in years, it is specified. All the values are approximated at one decimal

**Table 2:** Surgical details: type of surgical procedure adopted, type of suture and associated lesions

Authors and publication year	Arthroscopic inspection	Surgical procedure description	Type of suture	Associated lesions, (N)
LaPrade et al. 2005 [5]	Direct inspection PMFs + probing lateral meniscus	Open repair	Horizontal mattress non-absorbable 0 suture	0 (apparently normal MRI)
Kamiya et al. 2018 [7]	Direct inspection PMFs + probing lateral meniscus	Inside-out arthroscopic repair	Polyester non-absorbable sutures on a 10-inch straight and/or curved cutting needle. Average of 5.0 double-stacked vertical sutures	0
Zheng et al. 2021 [15]	A PMFs tear was identified by probing	All-inside arthroscopic repair	Suture hook technique (No. 2–0 non-absorbable suture)	ACL tear (27); PLC tear (7)
Keyhani et al. 2021 [8]	Direct inspection PMFs + probing lateral meniscus	All-inside arthroscopic repair	Suture hook with a PDS n.1 replaced with a fiber wire (n. 2). Vertical mattress suture repeated every 5–10 mm + 7 mm tunnel in the notch area to enhance healing	0
Di Vico et al. 2022 [11]	Direct inspection PMFs + probing lateral meniscus	All-inside arthroscopic repair	Two-to-three sutures placed on either side of the popliteal hiatus in a vertical fashion	Patients with associated lesions (8): ACL tear (5); MM tear (1); Chondral lesion of lateral femoral condyle (4)

*PMFs* popliteomeniscal fascicles; *N* number; *MRI* magnetic resonance imaging; *ACL* anterior cruciate ligament; *PLC* posterolateral corner; *MM* medial meniscus; *mm* millimeters

**Table 3:** Postoperative evaluation with symptoms and physical examination, functional outcome scores, complications, postoperative imaging and return to previous activity level

Authors and Publication year	Symptoms and physical examinations, (N)	Functional outcome scores Mean ± SD (range)						Complications, (N)	Postoperative imaging	Return to previous activity, (N)
		Lysholm score		Tegner Activity Scale		IKDC score				
		Pre-op	Post-op	Pre-op	Post-op	Pre-op	Post-op			
LaPrade et al. 2005 [5]	Resolution of symptoms for all the patients. Figure-4 negative for all the patients	–	–	–	–	–	–	–	–	2 Professional wrestlers returned to unrestricted activity and competition*
Kamiya et al. 2018 [7]	No locking and no pain for all the patients	72.0 (48–85)	97.8 (78–100)	4.6 (2–8)	4.7 (2–8)	–	–	0	Virtual load 3D-MRI 4 months postop: all lateral menisci had physiological motion	All patients returned to previous activity level without pain
Zheng et al. 2021 [15]	No locking; No snapping; Figure-4 test -; Lachman test-; Anterior drawer test -; Pivot shift test-; McMurray -	53.1 ± 0.8 (45–61)	85.7 ± 0.4 (82–90)	–	–	49.1 ± 1.1 (38–58)	83.7 ± 0.4 (79–88)	–	MRI: evidence of healing	All patients returned to normal work
Keyhani et al. 2021 [8]	No recurrence of locking, no need of reoperation for all the patients	63.5 ± 3.0	91.0 ± 2.0	–	–	58.5 ± 5.0	85.0 ± 3.0	No complications associated to arthroscopy. 1 patient sporadic pain 2 years after surgery which resolved	– (Reported as limitation of the study the lack of dynamic MRI after surgery)	All patients returned to the previous activity level
Di Vico et al. 2022 [11]	Good–Excellent (14); Fair-good (3) outcomes	56.7 ± 8.2 (40–68)	89.8 ± 3.2 (80–96)	2.9 ± 1.3 (0–5)	6.5 ± 2 (2–9)	60.2 ± 13.5 (27–92)	83.1 ± 12.0 (43–100)	0	MRI at 6 months postop: healing of the repaired PMFs fascicle in all the cases	Full return to pre-injury level of sports (13); Semi-professional to recreational activity (3); Change of sport activity (1)

^*^In the text, only the return to previous activity of these two patients is specified

-: Means the test is negative

*MRI* magnetic resonance imaging; *PMFs* popliteomeniscal fascicles; *Pre-op* preoperative; *Post-op* postoperative; *N* number; *IKDC score* International Knee Documentation Committee score; *SD* standard deviation

–: Not reported/not mentioned in the paper. All the values are approximated at one decimal

**Table 4:** Postoperative rehabilitation protocol

Authors and publication year	Rehabilitation protocol
LaPrade et al. 2005 [5]	Immobilization in full extension No WB for 6 weeks. During the 6 weeks: straight-leg raises in the immobilizer only and unrestricted active and passive knee motion 4 times a day. WB and progressive exercise at 6 weeks. Full return to activities at 4 months postoperatively
Kamiya et al. 2018 [7]	Open kinetic chain and ROM exercise immediately, full WB with semi-rigid knee extension brace on the day after surgery. Brace for 4 weeks. Squatting and sports activities at 12 weeks postoperatively
Zheng et al. 2021 [15]	Long knee brace with knee 0° flexion, non-weight-bearing ROM exercise with flexion limit of 90° from the 1st postoperative day up to 3 weeks postoperatively. Flexion > 90° from the 4th week after surgery, quadriceps exercise from 6th week. Running 3 months postoperatively
Keyhani et al. 2021 [8]	Initial 4 weeks of full-extension splint; then gradual range of motion to achieve 90° flexion over 8 weeks. Partial WB 2 weeks after surgery. Full WB 12 weeks after surgery. Return to pre-injury and normal sports activities after 6 months of rehabilitation
Di Vico et al. 2022 [11]	Initial 0–2 weeks after surgery: extension brace, partial WB, passive ROM 0–90° and single leg-raise. 2–4 weeks after surgery: brace removal and active-assisted ROM 0–90°. At 4th week: full WB and full passive ROM, closed kinetic chain with knee flexion 0–90°. Running at 3–4 months. Unrestricted return to sport 4–6 months

*WB* weight-bearing; *ROM* range of motion

**Fig. 3:**
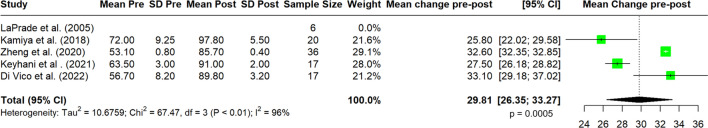
Forest plot. Comparison of Lysholm score results between preoperative and postoperative values. *SD* standard deviation; *CI* confidence interval; *p p* value

**Fig. 4:**
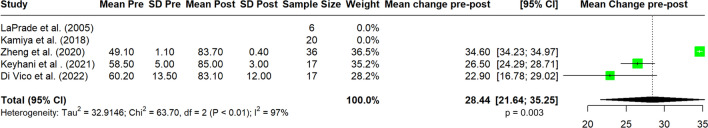
Forest plot. Comparison of subjective IKDC score results between preoperative and postoperative values. *SD* standard deviation; *CI* confidence interval; *p p* value

**Fig. 5:**
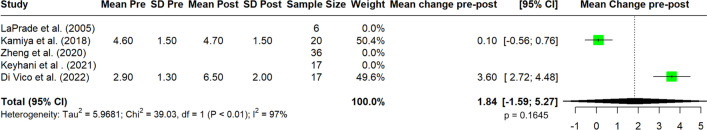
Forest plot. Comparison of Tegner Activity Scale results between preoperative and postoperative values. *SD* standard deviation; *CI* confidence interval; *p p* value

A meta-analysis of the Lysholm score, the Tegner Activity Scale and the subjective IKDC score was performed considering pre- and postoperative values. The Lysholm score and the subjective IKDC score showed a statistically significant difference suggesting improvement following the surgical procedure, p values 0.0005 and 0.003 respectively (Figs. 3, 4). The Tegner Activity Scale did not exhibit a statistically significant difference between pre and postoperative values, p value 0.1645 (Fig. 5).

## Discussion

The most important finding of this SR and meta-analysis was a statistically significant difference in the Lysholm score and subjective IKDC score between the preoperative and postoperative evaluation in symptomatic patients with diagnosed PMFs tears. In agreement with the recent literature, it promotes the surgical management of PMFs injuries, especially in young athletes, since surgery improves PROMs and it is resolutive for symptoms. Furthermore, this SR in line with the literature underlines the importance of directly visualizing PMFs tears arthroscopically with probing of the lateral meniscus as the gold standard in the diagnosis.

PMFs belong to the popliteal hiatus complex in knee PLC [1–5]. Despite a detailed and meticulous anatomical description of the PMFs, a quantitative description of the structural attachment with the relative length of the three PMFS is essential to avoid potential over-constraint of the mobile lateral meniscus during surgical repair [1–4, 15, 29]. PMFs tears are usually associated with other knee injuries, such as ACL and PLC injuries [1, 9, 11–13, 30]. Stäubli and Birrer described an overall PMFs tears of 13.1% in intact ACL knees, while in the acute and chronic ACL-deficient setting, the percentage raises to 57.5% and 50%, respectively [1]. LaPrade reported aiPMFs tears in 83% of the knees with grade III posterolateral complex injuries [12]. Thus, rarely PMFs occur as isolated lesions and PMFs injuries were described as one of the causes of hypermobile lateral meniscus [2, 4, 7–9].

In all the scenarios, the diagnosis of PMFs tears remains challenging, both from a clinical point of view and from an MRI investigation [4, 5, 9–11].

First, this pathological condition should be suspected in young athletes involved in sports with frequent changes of directions [9]. Patients included in this SR are aligned with the most recent literature since, apart from Kamiya et al. [7], whose patients have a broader range of ages, the other authors included patients younger than 50 years old [5, 8, 11, 15].

Symptoms were one of the indications for surgery since the surgical procedure was suggested to be resolutive, with no relevant complications associated [9]. From a clinical point of view, the main symptoms are locking or/and snapping and pain on the lateral aspect of the knee [4, 9, 11]. This clinical appearance was attributed to the altered physiologic motion of the lateral meniscus since the posterior portion moved forward with flexion and translated backward with extension [7, 8]. As described by Simonian et al. [2] as well as by Laprade and Konowalchuk [5], the meniscus could become trapped within the joint, also visualized in MRI [31].

To implement the diagnostic evaluation, LaPrade and Konowalchuk introduced the figure-4 position clinical test stressing the PLC and eliciting pain due to entrapment into the joint of the abnormal mobile lateral meniscus in case of PMFs tears [5]. This assessment test could be accomplished either preoperatively or postoperatively, and as noted in this SR, all the patients examined after surgery had a negative test [5, 15]. Regarding imaging, preoperative MRI is employed. However, LaPrade and Konowalchuk [5], as well as previously Simonian et al. [2], described a normal preoperative MRI, and only after a careful review of the MRI, they detected retrospectively the PMFs tears. Due to the interindividual variability of the fascicles, Sakai et al. tried to investigate the most suitable MRI parameters to detect PMFs [10]. They reported that proton density-weighted images of 3-mm slice thickness and T2-weighted images with 45° oblique coronal views should be done [10]. D'Addona et al. [9], along with Di Vico et al. [11], proposed the use of the sagittal plane and T2 sequences to detect these lesions. Although not routinely utilized in the diagnostic evaluation, Kamiya et al. utilized pre- and postoperatively virtual load 3D-MRI to analyze meniscal motion [7]. One crucial aspect already introduced in the previous SR and further underlined in this study is the importance of performing MRI postoperatively. In all the studies performing postoperative imaging assessment, MRI showed healing of lesions at the final follow-up [7, 11, 15].

**Fig. 6:**
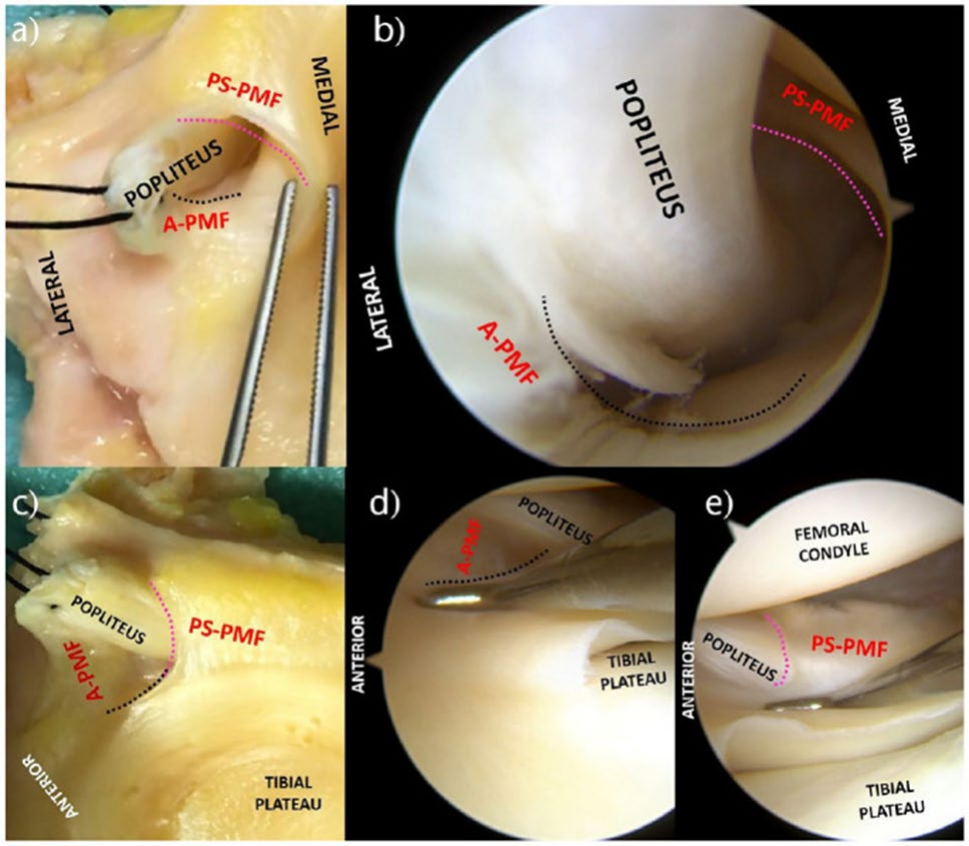
Anatomical specimen of a right lateral tibial plateau at the level of the popliteal hiatus (**a**) and arthroscopic view of a right knee ‘popliteus tunnel’, with extended knee and the scope in the lateral recess through the antero-lateral portal (**b**). It is possible to appreciate the integrity of the popliteus tendon, the free margin of the PS-PMF (purple dotted line) and the outline of the A-PMF (black dotted line). Anatomical specimen of a right lateral tibial plateau (**c**) and arthroscopic view of a right knee lateral meniscus (**d**, **e**). Pushing down the meniscus with a probe, it is possible to expose and examine the integrity of the PS-PMF (purple dotted line) posteriorly to the popliteus tendon, and of the A-PMF (black dotted line) anteriorly. *PS-PMF* postero-superior popliteomeniscal fascicle; *A-PMF* anterior popliteomeniscal fascicle. The source is published under a Creative Commons License from Grassi A. et al [4]

**Fig. 7:**
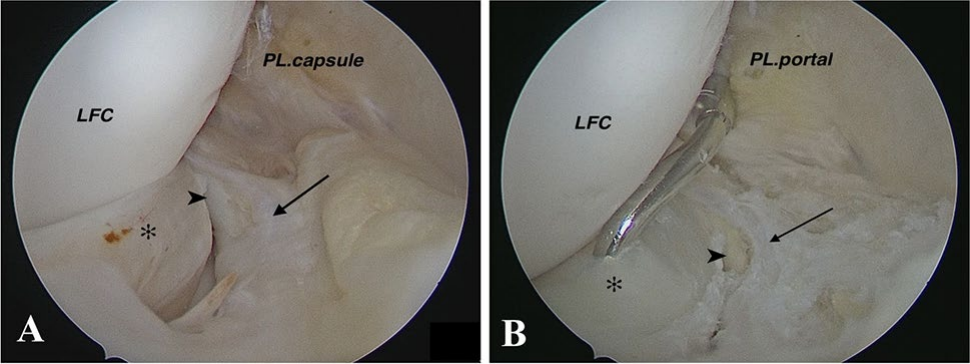
Right knee arthroscopy: **A** Posteromedial transseptal view with a 30-degree lens that shows popliteomeniscal fascicle tear **B** repair by using suture hook technique from posterolateral portal. Asterisk: Lateral meniscus; Arrow: Posterosuperior popliteomeniscal tear; Arrow head: Popliteus tendon. The source is published under a Creative Commons License from Keyhani S. et al [8]

When a patient has symptoms, and the clinical suspicion is high, the gold standard for the diagnosis, already well-established in the literature, is the direct arthroscopic visualization of the PMFs tears with probing of the lateral meniscus to assess its mobility [4, 5, 9, 14] (Fig. 6). When the diagnosis of PMFs is confirmed, symptomatic patients should be directed to surgery since it is associated with the resolution of symptoms [4, 5, 9]. As described by D' Addona et al. in 2021, time is not resolutive of PMFs tears since the repetitive meniscal motion prevents scar tissue formation [9]. Guimaraes JB et al. [32] showed higher cartilage damage in the lateral femoral compartment over two years in subjects with PMFs and ACL injuries concerning ACL deficiencies alone. In agreement with D'Addona et al. [9], surgery was a safe procedure with few associated complications. Nonetheless, in literature, controversy remains about the most appropriate surgical strategy to be adopted, regarding either the open or arthroscopic approach and the suturing technique [9]. Despite LaPrade and Konowalchuk performing an open repair, they postulated the use of arthroscopy to restore the integrity of PMFs to be a successful option [5]. Currently, there seems to be a trend in favor of an arthroscopic technique [8, 11, 15] (Fig. 7).

This SR and meta-analysis have several strengths. The most innovative result of this meta-analysis is that it reports not only the complete resolution of the clinical symptoms after surgery, but it analyzes the PROMs pre- and postoperatively, since in the previous systematic review by D’Addona et al. [9] only Kamjya et al. [7] analyzed results with subjective knee scores. Pre- and postoperative outcomes using internationally validated PROMs such as the Lysholm score, the Tegner Activity Scale, and the subjective IKDC score were analyzed. However, not all the studies described all the PROMs, with four studies reporting the Lysholm score [7, 8, 11, 15], two studies the Tegner Activity Scale [7, 11], and three studies the subjective IKDC score [8, 11, 15]. Furthermore, in three studies, the postoperative lesion healing was confirmed through an MRI at the final follow-up [7, 11, 15].

Nevertheless, limitations should be analyzed. In the literature, there are numerous detailed anatomical reports of the PMFs and their tears, especially case reports, but few case series are present with all the inclusion criteria adopted in this SR. For this reason, only five studies were included in the analysis, and all were IV as LoE. Moreover, the number of patients included in each study was low. Although a statistically significant improvement of the Lysholm and the subjective IKDC score postoperatively was detected, the I2 was > 90% in all the scores analyzed. A potential explanation could have been the heterogeneity of the studies, especially regarding the follow-up time and the duration of symptoms before surgery. More homogenous studies could improve the validity of the data. Furthermore, the heterogeneity in the surgical procedures embraced could create possible bias. However, due to the scanty clinical studies concerning PMFs tears, all the studies were included to increase the number of patients and the firmness of the meta-analysis. Finally, the limited number of studies hindered a quantitative analysis comparing the potential superiority of one technique over the other. Moreover, some studies' low quantity of data and incomplete reporting of variables precluded the execution of specific sensitivity analysis, meta-regression analysis, and application of the GRADE approach. Further high-quality studies and RCTs will be necessary to settle the best surgical procedure, either the open or the arthroscopic approach as well as the most appropriate suturing technique.

## Conclusion

This SR and meta-analysis demonstrated a statistically significant improvement in the Lysholm score and subjective IKDC score after surgery for PMFs tears. The diagnosis gold standard is the direct arthroscopic visualization of the PMFs tears with probing of the lateral meniscus to assess its mobility. Symptomatic young patients should be directed to a surgical solution since surgery appears to be resolutive for symptoms. Controversy remains about the most appropriate surgical strategy to be embraced, either the open or the arthroscopic approach and the suturing technique, with most current literature adopting an arthroscopic procedure.

## Data Availability

The dataset analyzed in this study is available from the corresponding author on reasonable request.
